# Worse survival of hepatocellular cancer patients with membranous insulin receptor overexpression

**DOI:** 10.1038/s41598-025-85350-2

**Published:** 2025-01-07

**Authors:** Steffen Markus Heckl, Carolin Schneider, Lukas Kercher, Hans-Michael Behrens, Jan-Paul Gundlach, Alexander Bernsmeier, Stephan Schmidt, Sandra Krüger, Felix Braun, Rainer Günther, Thomas Becker, Stefan Schreiber, Christoph Röcken

**Affiliations:** 1 Department of Pathology, Christian-Albrechts-University, University Hospital Schleswig-Holstein, 24105 Kiel, Germany; 2 Department of Internal Medicine II, Christian-Albrechts-University, University Hospital Schleswig-Holstein, 24105 Kiel, Germany; 3 Department of Internal Medicine I, Christian-Albrechts-University, University Hospital Schleswig-Holstein, 24105 Kiel, Germany; 4 Department of General, Visceral, Thoracic, Transplant and Pediatric Surgery, Christian-Albrechts-University, University Hospital Schleswig-Holstein, 24105 Kiel, Germany

**Keywords:** Hepatocellular cancer, IGF1 receptor, Insulin receptor, Prognosis, Sorafenib, Hepatocellular carcinoma, Predictive markers, Prognostic markers

## Abstract

Hepatocellular cancer (HCC) therapy is in need for an ideal companion diagnostic. Preclinical experimental studies have identified the insulin receptor (IR) and its synergistic counterpart, the IGF1 receptor (IGF1R), as relevant in HCC development, and the ligands IGF1 and IGF2 have been found to be elevated in HCC. This study aimed to bridge the gap to the clinical setting and explore whether the IR or the IGF1R would be of prognostic significance and would be associated with clinicopathologic parameters in HCC patients. In our retrospective cohort study located at the University Hospital Schleswig-Holstein, Campus Kiel, Germany, HCC samples of 139 patients were examined for IR and IGF1R expression by immunohistochemistry. A HistoScore was correlated with clinicopathological characteristics and survival. IR overexpression was frequently observed and was associated with clinicopathological parameters and survival. Membranous IR expression was associated with worse tumor specific survival (*p* = 0.043). Intriguingly, membranous IR expression was associated with worse tumor specific survival (*p* = 0.017) in the subgroup of patients undergoing sorafenib therapy. IGF1R expression was not associated with survival. In conclusion, our results suggest that membranous IR expression plays a role in HCC prognosis and treatment resistance, inspiring future validation as a potential companion diagnostic in HCC.

**Supplementary Information:**

The online version contains supplementary material available at 10.1038/s41598-025-85350-2.

## Introduction

Hepatocellular cancer (HCC) is the third leading cause of cancer-related death worldwide^1^. The global prevalence of HCC has been increasing over time^2^. However, the underlying etiology of HCC development is currently undergoing a steady shift from predominantly viral to increasingly non-viral causes^1,2^. Dedicated hepatitis B virus vaccination programs and the development of effective antiviral drugs against hepatitis C virus infection have led to a steady decline in virus-associated HCC incidence rates^2^. Although chronic viral hepatitis is still the leading cause of HCC worldwide, non-viral causes such as metabolic dysfunction associated steatotic liver disease (MASLD) are increasing dramatically, resulting in a rising incidence of non-viral HCC worldwide^1,2^.

HCCs are regularly diagnosed at an advanced stage, which contributes to its detrimental prognosis^3^. During the recent years, therapeutic options for patients with advanced HCC have expanded. Prognostic biomarkers are required more than ever for an improvement and personalization of therapeutic algorithms. Until now, no companion diagnostic has been apt to fly in HCC therapy, as prior candidates have never completely fulfilled the mandatory “pre-flight check-list”^3^.

In search of prognostic biomarkers, we identified the insulin receptor (IR) to be overexpressed in cancer cells (CC-IR) and vasculature (VIR) of gastrointestinal cancer entities^4–7^. IR expression was associated with clinicopathological parameters and survival. For the IR, two isoforms exist: IR isoform B (IR-B) conveys the commonly known metabolic effects of the hormone insulin. IR isoform A (IR-A) on the other hand induces proliferative effects and is mainly expressed in embryonic as well as in cancer tissue^4,8^. IR signaling has to be seen in a context with the insulin like growth factor 1 receptor (IGF1R), as both are known to form the synergetic IR-/IGF1R-axis: The IR-A as well as the IGF1R, are known to be stimulated by IGF1 and IGF2^8^. Both hormones have been found to be elevated in HCC specimens^9^. IR-A has been reported to be the predominantly expressed IR isoform in HCC in contrast to adjacent noncancerous liver tissue^10^. While there is preclinical evidence that IR and IGF1R may play a role in early hepatocarcinogenesis^11^ and that insulin receptor substrates may play a role in HCC progression^12^, real-world clinical data are needed.

We therefore wanted to know, if the IR or the IGF1R would be of prognostic significance and would be associated with clinicopathological patient characteristics in HCC. We further wanted to evaluate, if the IR or the IGF1R expression status might fulfill the prerequisites needed to contribute to the prediction of HCC therapy outcomes. We tested the following hypotheses:

(I) HCCs express the IR in cancer cells (CC-IR) and cancer vasculature (VIR). (II) IR expression in HCC correlates with clinicopathological patient characteristics, including survival. (III) The expression of IGF1R in HCC is associated with clinicopathological patient characteristics and survival and (IV) is linked to the expression of the IR. (V) The IR and the IGF1R are differentially expressed in hepatic adenomas (HCA) when compared with healthy liver tissue.

## Results

### Characteristics of the study populatio

The clinicopathological patient characteristics of the HCC cohort are summarized in Tables 1 and 2. 139 patients fulfilled all study criteria and were included in the study. The median age at the time of diagnosis was 65 years. The study cohort was comprised of 37 (26.6%) female and of 102 (73.4%) male patients. 33 patients (23.7%) had more than one HCC lesion; the maximal number of lesions in a single patient’s liver being 12. In these cases the lesion with the largest diameter was chosen and included in our study. In 106 patients (76.3%) the HCC presented itself as a single lesion. The median tumor diameter was 3.2 cm. Systemic therapy included palliative therapy regimens such as with tyrosine kinase inhibitors (e.g. sorafenib), or chemotherapeutic drugs (cisplatin and doxorubicin or gemcitabine and oxaliplatin). As the study cohort was comprised of HCC specimens from the years 2004 to 2018, it did not include patients with immunotherapy regimens.

**Table 1:** Correlation between clinicopathological patient characteristics and the expression of the insulin receptor (IR) in cancer vessels and cells, respectively.

Clinicopathological characteristics		Total		IR vascular expression				Cytoplasmic IR expression				Membranous IR expression			
				Low HScore ≤ 120		High HScore > 120		Low HScore ≤ 39		High HScore > 39		Low HScore < 35		High HScore ≥ 35	
		n	(%)	n	(%)	n	(%)	n	(%)	n	(%)	n	(%)	n	(%)
**Sex**	n p-value^†^	*n* = 139		*n* = 139			*p* = 0.448	*n* = 139			*p* = 0.702	*n* = 139			*p* = 0.849
male		102	(73.4)	50	(49.0)	52	(51.0)	50	(49.0)	52	(51.0)	50	(49.0)	52	(51.0)
female		37	(26.6)	21	(56.8)	16	(43.2)	20	(54.1)	17	(45.9)	19	(51.4)	18	(48.6)
**Liver cirrhosis**	n p-value^†^	*n* = 139		*n* = 139			*p* = 0.853	*n* = 139			*p* = 0.137	*n* = 139			*p* = 0.711
cirrhotic		99	(71.2)	50	(50.5)	49	(49.5)	54	(54.5)	45	(45.5)	48	(48.5)	51	(51.5)
non-cirrhotic		40	(28.8)	21	(52.5)	19	(47.5)	16	(40.0)	24	(60.0)	21	(52.5)	19	(47.5)
**Diabetes mellitus**	n p-value^†^	*n* = 139		*n* = 139			*p* = 0.862	*n* = 139			*p* = 1.000	*n* = 139			*p* = 1.000
diabetic		55	(39.6)	29	(52.7)	26	(47.3)	28	(50.9)	27	(49.1)	27	(49.1)	28	(50.9)
non-diabetic		84	(60.4)	42	(50.0)	42	(50.0)	42	(50.0)	42	(50.0)	42	(50.0)	42	(50.0)
**Hepatitis**	n p-value^†^	*n* = 139		*n* = 139			*p* = 1.000	*n* = 139			*p* = 0.373	*n* = 139			*p* = 0.156
Yes		48	(34.5)	25	(52.1)	23	(47.9)	27	(56.3)	21	(43.8)	28	(58.3)	20	(41.7)
No		91	(65.5)	46	(50.0)	45	(49.5)	43	(47.3)	48	(52.7)	41	(45.1)	50	(54.9)
**MASLD**	n p-value^†^	*n* = 139		*n* = 139			*p* = 1.000	*n* = 139			*p* = 0.586	*n* = 139			0.586
Yes		14	(10.1)	7	(50.0)	7	(50.0)	6	(42.9)	8	(57.1)	8	(57.1)	6	(42.9)
No		125	(89.9)	64	(51.2)	61	(48.8)	64	(51.2)	61	(48.8)	61	(48.8)	64	(51.2)
**Child Pugh**	n p-value^‡^	*n* = 139		*n* = 139			*p* = 0.797	*n* = 139			*p* = 0.194	*n* = 139			*p* = 0.946
non-cirrhotic		40	(28.8)	21	(52.5)	19	(47.5)	16	(40.0)	24	(60.0)	21	(52.5)	19	(47.5)
Stadium A		58	(41.7)	29	(50.0)	29	(50.0)	32	(55.2)	26	(44.8)	27	(46.6)	31	(53.4)
Stadium B		30	(21.6)	17	(56.7)	13	(43.3)	15	(50.0)	15	(50.0)	15	(50.0)	15	(50.0)
Stadium C		11	(7.9)	4	(36.4)	7	(63.6)	7	(63.6)	4	(36.4)	6	(54.5)	5	(45.5)
**BCLC**	n p-value^‡^	*n* = 139		*n* = 139			*p* = 0.752	*n* = 139			*p* = 0.956	*n* = 139			*p* = 0.711
A1-A2		25	(18.0)	12	(48.0)	13	(52.0)	11	(44.0)	14	(56.0)	13	(52.0)	12	(48.0)
A3		35	(25.2)	19	(54.3)	16	(45.7)	20	(57.1)	15	(42.9)	17	(48.6)	18	(51.4)
A4		7	(5.0)	2	(28.6)	5	(71.4)	4	(57.1)	3	(42.9)	4	(57.1)	3	(42.9)
B		55	(39.6)	33	(60.0)	22	(40.0)	27	(49.1)	28	(50.9)	28	(50.9)	27	(49.1)
C		6	(4.3)	1	(16.7)	5	(83.3)	1	16.7)	5	(83.3)	1	(16.7)	5	(83.3)
D		11	(7.9)	4	36.4)	7	(63.6)	7	(63.6)	4	(36.4)	6	(54.5)	5	(45.5)
**Alcoholic liver** **cirrhosis**	n p-value^†^	*n* = 139		*n* = 139			*p* = 0.139	*n* = 139			*p* = 0.464	*n* = 139			*p* = 0.357
Yes		42	(30.2)	17	(40.5)	25	(59.5)	19	(45.2)	23	(54.8)	18	(42.9)	24	(57.1)
No		97	(69.8)	54	(55.7)	43	(44.3)	51	(52.6)	46	(47.4)	51	(52.6)	46	(47.4)
**Solitary tumor**	n p-value^†^	*n* = 139		*n* = 139			*p* = 0.842	*n* = 139			*p* = 0.844	*n* = 139			*p* = 0.691
Yes		106	(76.3)	55	(51.9)	51	(48.1)	54	(50.9)	52	(49.1)	54	(50.9)	52	(49.1)
No		33	(23.7)	16	(48.5)	17	(51.5)	16	(48.5)	17	(51.5)	15	(45.5)	18	(54.5)
**Multifocal**	n p-value^†^	*n* = 139		*n* = 139			*p* = 0.842	*n* = 139			*p* = 0.844	*n* = 139			*p* = 0.691
Yes		33	(23.7)	16	(48.5)	17	(51.5)	16	(48.5)	17	(51.5)	15	(45.5)	18	(54.5)
No		106	(76.3)	55	(51.9)	51	(48.1)	54	(50.9)	52	(49.1)	54	(50.9)	52	(49.1)
**Inside Milan**	n p-value^†^	*n* = 139		*n* = 139			*p* = 0.496	*n* = 139			*p* = 0.497	*n* = 139			*p* = 0.865
Yes		77	(55.4)	37	(48.1)	40	(51.9)	41	(53.2)	36	(46.8)	39	(50.6)	38	(49.4)
No		62	(44.6)	34	(54.8)	28	(45.2)	29	(46.8)	33	(53.2)	30	(48.4)	32	(51.6)
**Inside UCSF**	n p-value^†^	*n* = 139		*n* = 139			*p* = 1.000	*n* = 139			*p* = 0.190	*n* = 139			*p* = 1.000
Yes		100	(71.9)	51	(51.0)	49	(49.0)	54	(54.0)	46	(46.0)	50	(50.0)	50	(50.0)
No		39	(28.1)	20	(51.3)	19	(48.7)	16	(41.0)	23	(59.0)	19	(48.7)	20	(51.3)
**Vascular invasion status**	n p-value^†^	*n* = 100		*n* = 100			*p* = 0.582	*n* = 100			*p* = 1.000	*n* = 100			*p* = 0.400
pV0		85	(85.0)	42	(49.4)	43	(50.6)	47	(55.3)	38	(44.7)	51	(60.0)	34	(40.0)
pV+ (pV1, pV2)		15	(15.0)	6	(40.0)	9	(60.0)	8	(53.3)	7	(46.7)	7	(46.7)	8	(53.3)
**AFP positive**	n p-value^†^	*n* = 139		*n* = 139			*p* = 0.498	*n* = 139			*p* = 0.734	*n* = 139			*p* = 0.611
Yes		76	(54.7)	41	(53.9)	35	(46.1)	37	(48.7)	39	(51.3)	36	(47.4)	40	(52.6)
No		63	(45.3)	30	(47.6)	33	(52.4)	33	(52.4)	30	(47.6)	33	(52.4)	30	(47.6)
**AFP positive max. 400**	n p-value^†^	*n* = 139		*n* = 139			*p* = 0.489	*n* = 139			*p* = 0362	*n* = 139			*p* = 0.649
low ( < = 400)		117	(84.2)	58	(49.6)	59	(50.4)	61	(52.1)	56	(47.9)	57	(48.7)	60	(51.3)
high (> 400)		22	(15.8)	13	(59.1)	9	(40.9)	9	(40.9)	13	(59.1)	12	(54.5)	10	(45.5)
**R status**	n p-value^†^	*n* = 118		*n* = 118			*p* = 0.516	*n* = 118			*p* = 1.000	*n* = 118			*p* = 0.807
R0		112	(94.9)	58	(51.8)	54	(48.2)	61	(54.5)	51	(45.5)	63	(56.3)	49	(43.8)
R1		5	(4.2)	2	(40.0)	3	(60.0)	3	(60.0)	2	(40.0)	3	(60.0)	2	(40.0)
Rx		1	(0.8)	0	(0.0)	1	(100.0)	1	(100.0)	0	(0.0)	0	(0.0)	1	(100.0)
**Liver transplantation**	n p-value^†^	*n* = 139		*n* = 139			*p* = 0.612	*n* = 139			*p* = 0.310	*n* = 139			*p* = 0.128
Yes		67	(48.2)	36	(53.7)	31	(46.3)	37	(55.2)	30	(44.8)	38	(56.7)	29	(43.3)
No		72	(51.8)	35	(48.6)	37	(51.4)	33	(45.8)	39	(54.2)	31	(43.1)	41	(56.9)
**Resection**	n p-value^†^	*n* = 139		*n* = 139			*p* = 0.612	*n* = 139			*p* = 0.398	*n* = 139			*p* = 0.042*
Yes		68	(48.9)	33	(48.5)	35	(51.5)	37	(54.4)	31	(45.6)	40	(58.8)	28	(41.2)
No		71	(51.1)	38	(53.5)	33	(46.5)	33	(46.5)	31	(53.5)	29	(40.8)	42	(59.2)
**TACE**	n p-value^†^	*n* = 139		*n* = 139			*p* = 1.000	*n* = 139			*p* = 1.000	*n* = 139			*p* = 0.735
Yes		65	(46.8)	33	(50.8)	32	(49.2)	33	(50.8)	32	(49.2)	31	(47.7)	34	(52.3)
No		74	(53.2)	38	(51.4)	36	(48.6)	37	(50.0)	37	(50.0)	38	(51.4)	36	(48.6)
**SIRT**	n p-value^†^	*n* = 139		*n* = 139			*p* = 0.359	*n* = 139			*p* = 0.366	*n* = 139			*p* = 0.620
Yes		4	(2.9)	1	(25.0)	3	(75.0)	1	(25.0)	3	(75.0)	1	(25.0)	3	(75.0)
No		135	(97.1)	70	(51.9)	65	(48.1)	69	(51.1)	66	(48.9)	68	(50.4)	30	(49.6)
**PEI**	n p-value^†^	*n* = 139		*n* = 139			*p* = 0.209	*n* = 139			*p* = 0.208	*n* = 139			*p* = 1.000
Yes		11	(7.9)	8	(72.7)	3	(27.3)	8	(72.7)	3	(27.3)	5	(45.5)	6	(54.5)
No		128	(92.1)	63	(49.2)	65	(50.8)	62	(48.4)	66	(51.6)	64	(50.0)	64	(50.0)
**Systemic therapy**	n p-value^†^	*n* = 139		*n* = 139			*p* = 0.824	*n* = 139			*p* = 0.076	*n* = 139			*p* = 0.012*
Yes		24	(17.3)	13	(54.2)	11	(45.8)	8	(33.3)	16	(66.7)	6	(25.0)	18	(75.0)
No		115	(82.7)	58	(50.4)	57	(49.6)	62	(53.9)	53	(46.1)	63	(54.8)	52	(45.2)
**BMI**	n p-value^†^	*n* = 139		*n* = 139			*p* = 0.499	*n* = 139			*p* = 0.128	*n* = 139			*p* = 0.499
Low (≤ BMI 25.82 Kg/m2)		68	(48.9)	37	(54.4)	31	(45.6)	39	(57.4)	29	(42.6)	36	(52.9)	32	(47.1)
High (> BMI 25.82 Kg/m2)		71	(51.1)	34	(47.9)	37	(52.1)	31	(43.7)	40	(56.3)	33	(46.5)	38	(53.5)
**Size of the largest lesion**	n p-value^†^	*n* = 125		*n* = 125			*p* = 0.285	*n* = 125			*p* = 0.858	*n* = 125			*p* = 0.209
Low (≤ median size 3.2 cm)		63	(50.4)	29	(46.0)	34	(54.0)	35	(55.6)	28	(44.4)	31	(49.2)	32	(50.8)
High (> median size 3.2 cm)		62	(49.6)	35	(56.5)	27	(43.5)	33	(53.2)	29	(46.8)	38	(61.3)	24	(38.7)
**Overall Survival [Months]**	n p-value^§^	*n* = 133		*n* = 133			*p* = 0.580	*n* = 133			*p* = 0.412	*n* = 133			*p* = 0.055
Total / Events / Censored		133 / 50 / 83		70 / 24 / 46		63 / 26 / 37		67 / 23 / 44		66 / 27 / 39		66 / 21 / 45		67 / 29 / 38	
Median Survival		62.0		70.9		58.7		–		62.0		77.1		46.0	
95% C.I. (standard error)		49.9–74.1 (+-6.2)		–		41.3–76.1 (+-8.9)		–		44.2–79.7 (+-9.1)		53.7-100.5 (+-11.9)		25.5–66.4 (+-10.4)	
**Tumor Specific Survival [Months]**	n p-value^§^	*n* = 126		*n* = 126			*p* = 0.351	*n* = 126			*p* = 0.229	*n* = 126			*p* = 0.043*
Total / Events / Censored		126 / 21 / 105		68 / 9 / 59		58 / 12 / 46		63 / 8 / 55		63 / 13 / 50		63 / 7 / 56		63 / 14 / 49	
Median Survival		n.c.		n.c.		n.c.		n.c.		n.c.		n.c.		n.c.	
95% C.I. (standard error)		n.c.		n.c.		n.c.		n.c.		n.c.		n.c.		n.c.	
**Sorafenib Overall Survival [Months]**	n p-value^§^	*n* = 18		*n* = 18			*p* = 0.066	*n* = 18			*p* = 0.927	*n* = 18			*p* = 0.066
Total / Events / Censored		n.c.		8 / 3 / 5		10 / 6 / 4		6 / 3 / 3		12 / 6 / 6		5 / 2 / 3		13 / 7 / 6	
Median Survival		n.c.		70.9		33.4		11.0		33.4		70.9		33.4	
95% C.I. (standard error)		n.c.		1.1-140.7 (+-35.6)		10.6–56.2 (+-11.6)		n.c.		11.7–55.2 (+-11.1)		0.0-159.2 (+-45.1)		18.1–48.7 (+-7.8)	
**Sorafenib Tumor Specific Survival [Months]**	n p-value^§^	*n* = 17		*n* = 17			*p* = 0.192	*n* = 17			*p* = 0.987	*n* = 17			*p* = 0.017*
Total / Events / Censored		n.c.		8 / 2 / 6		9 / 4 / 5		5 / 2 / 3		12 / 4 / 8		4 / 0 / 4		13 / 6 / 7	
Median Survival		n.c.		n.c.		33.4		n.c.		48.0		n.c.		33.4	
95% C.I. (standard error)		n.c.		n.c.		1.2–65.7 (+-16.5)		n.c.		25.5–70.4 (+-11.5)		n.c.		18.3–48.6 (+-7.7)	

† Fisher’s exact test, ‡ Kendall’s tau test, § Log-rank test. * p values having lost significance according to the Siemes (Benjamini-Hochberg) procedure for multiple testing. Abbreviations: n.c., not calculated; MASLD, metabolic dysfunction associated steatotic liver disease; BCLC, Barcelona Clinic Liver Cancer staging system; UCSF, University of California San Francisco criteria; AFP positive max. 400, AFP values below or above 400 ng/ml; TACE, transarterial chemoembolisation; SIRT, Selective Internal Radiation Therapy; PEI, percutaneous ethanol injection.

**Table 2:** Correlation between clinicopathological patient characteristics and the expression of the insulin-like growth factor receptor 1 (IGF1R) in cancer cells.

Clinicopathological characteristics		Total		Cytoplasmic IGF1R expression				Membranous IGF1R expression			
				Low HScore = 0		High HScore > 0		Low HScore = 0		High HScore > 0	
		n	(%)	n	(%)	n	(%)	n	(%)	n	(%)
**Sex**	n p-value^†^	*n* = 139		*n* = 139			*p* = 0.184	*n* = 139			*p* = 0.289
male		102	(73.4)	90	(88.2)	12	(11.8)	93	(91.2)	9	(8.8)
female		37	(26.6)	36	(97.3)	1	(2.7)	36	(97.3)	1	(2.7)
**Liver cirrhosis**	n p-value^†^	*n* = 139		*n* = 139			*p* = 0.347	*n* = 139			*p* = 0.724
cirrhotic		99	(71.2)	88	(88.9)	11	(11.1)	91	(91.9)	8	(8.1)
non-cirrhotic		40	(28.8)	38	(95.0)	2	(5.0)	38	(95.0)	2	(5.0)
**Diabetes mellitus**	n p-value^†^	*n* = 139		*n* = 139			*p* = 0.034*	*n* = 139			*p* = 0.051
diabetic		55	(39.6)	46	(83.6)	9	(16.4)	48	(87.3)	7	(12.7)
non-diabetic		84	(60.4)	80	(95.2)	4	(4.8)	81	(96.4)	3	(3.6)
**Hepatitis**	n p-value^†^	*n* = 139		*n* = 139			*p* = 0.137	*n* = 139			*p* = 0.737
Yes		48	(34.5)	41	(85.4)	7	(14.6)	44	(91.7)	4	(8.3)
No		91	(65.5)	85	(93.4)	6	(6.6)	85	(93.4)	6	(6.6)
**MASLD**	n p-value^†^	*n* = 139		*n* = 139			*p* = 0.621	*n* = 139			*p* = 0.265
Yes		14	(10.1)	12	(85.7)	2	(14.3)	12	(85.7)	2	(14.3)
No		125	(89.9)	114	(91.2)	11	(8.8)	117	(93.6)	8	(6.4)
**Child Pugh**	n p-value^‡^	*n* = 139		*n* = 139			*p* = 0.375	*n* = 139			*p* = 0.922
non-cirrhotic		40	(28.8)	38	(95.0)	2	(5.0)	38	(95.0)	2	(5.0)
Stadium A		58	(41.7)	52	(89.7)	6	(10.3)	52	(89.7)	6	(10.3)
Stadium B		30	(21.6)	25	(83.3)	5	(16.7)	28	(93.3)	2	(6.7)
Stadium C		11	(7.9)	11	(100.0)	0	(0.0)	11	(100.0)	0	(0.0)
**BCLC**	n p-value^‡^	*n* = 139		*n* = 139			*p* = 0.176	*n* = 139			*p* = 0.569
A1-A2		25	(18.0)	22	(88.0)	3	(12.0)	23	(92.0)	2	(8.0)
A3		35	(25.2)	30	(85.7)	5	(14.3)	32	(91.4)	3	(8.6)
A4		7	(5.0)	7	(100.0)	0	(0.0)	7	(100.0)	0	(0.0)
B		55	(39.6)	50	(90.9)	5	(9.1)	50	(90.9)	5	(9.1)
C		6	(4.3)	6	(100.0)	0	(0.0)	6	(100.0)	0	(0.0)
D		11	(7.9)	11	(100.0)	0	(0.0)	11	(100.0)	0	(0.0)
**Alcoholic liver cirrhosis**	n p-value^†^	*n* = 139		*n* = 139			*p* = 0.754	*n* = 139			*p* = 1.000
Yes		42	(30.2)	39	(92.9)	3	(7.1)	39	(92.9)	3	(7.1)
No		97	(69.8)	87	(89.7)	10	(10.3)	90	(92.8)	7	(7.2)
**Solitary tumor**	n p-value^†^	*n* = 139		*n* = 139			*p* = 0.733	*n* = 139			*p* = 1.000
Yes		106	(76.3)	95	(89.6)	11	(10.4)	98	(92.5)	8	(7.5)
No		33	(23.7)	31	(93.9)	2	(6.1)	31	(93.9)	2	(6.1)
**Multifocal**	n p-value^†^	*n* = 139		*n* = 139			*p* = 0.733	*n* = 139			*p* = 1.000
Yes		33	(23.7)	31	(93.9)	2	(6.1)	31	(93.9)	2	(6.1)
No		106	(76.3)	95	(89.6)	11	(10.4)	98	(92.5)	8	(7.5)
**Inside Milan**	n p-value^†^	*n* = 139		*n* = 139			*p* = 0.773	*n* = 139			*p* = 0.752
Yes		77	(55.4)	69	(89.6)	8	(10.4)	72	(93.5)	5	(6.5)
No		62	(44.6)	57	(91.9)	5	(8.1)	57	(91.9)	5	(8.1)
**Inside UCSF**	n p-value^†^	*n* = 139		*n* = 139			*p* = 1.000	*n* = 139			*p* = 1.000
Yes		100	(71.9)	90	(90.0)	10	(10.0)	93	(93.0)	7	(7.0)
No		39	(28.1)	36	(92.3)	3	(7.7)	36	(92.3)	3	(7.7)
**Vascular invasion status**	n p-value^†^	*n* = 100		*n* = 100			*p* = 0.363	*n* = 100			*p* = 0.132
pV0		85	(85.0)	77	(90.6)	8	(9.4)	79	(92.9)	6	(7.1)
pV+ (pV1, pV2)		15	(15.0)	12	(80.0)	3	(20.0)	12	(80.0)	3	(20.0)
**AFP positive**	n p-value^†^	*n* = 139		*n* = 139			*p* = 0.037*	*n* = 139			*p* = 0.112
Yes		76	(54.7)	65	(85.5)	11	(14.5)	68	(89.5)	8	(10.5)
No		63	(45.3)	61	(96.8)	2	(3.2)	61	(96.8)	2	(3.2)
**AFP positive max. 400**	n p-value^†^	*n* = 139		*n* = 139			0.433	*n* = 139			*p* = 0.659
low ( < = 400)		117	(84.2)	107	(91.5)	10	(8.5)	109	(93.2)	8	(6.8)
high (> 400)		22	(15.8)	19	(86.4)	3	(13.6)	20	(90.9)	2	(9.1)
**R status**	n p-value^†^	*n* = 118		*n* = 118			*p* = 1.000	*n* = 118			*p* = 1.000
R0		112	(94.9)	100	(89.3)	12	(10.7)	103	(92.0)	9	(8.0)
R1		5	(4.2)	5	(100.0)	0	(0.0)	5	(100.0)	0	(0.0)
Rx		1	(0.8)	1	(100.0)	0	(0.0)	1	(100.0)	0	(0.0)
**Liver transplantation**	n p-value^†^	*n* = 139		*n* = 139			*p* = 1.000	*n* = 139			*p* = 0.329
Yes		67	(48.2)	61	(91.0)	6	(9.0)	64	(95.5)	3	(4.5)
No		72	(51.8)	65	(90.3)	7	(9.7)	65	(90.3)	7	(9.7)
**Resection**	n p-value^†^	*n* = 139		*n* = 139			p = o.776	*n* = 139			*p* = 0.526
Yes		68	(48.9)	61	(89.7)	7	(10.3)	62	(91.2)	6	(8.8)
No		71	(51.1)	65	(91.5)	6	(8.5)	67	(94.4)	4	(5.6)
**TACE**	n p-value^†^	*n* = 139		*n* = 139			*p* = 0.382	*n* = 139			*p* = 0.515
Yes		65	(46.8)	57	(87.7)	8	(12.3)	59	(90.8)	6	(9.2)
No		74	(53.2)	69	(93.2)	5	(6.8)	70	(94.6)	4	(5.4)
**SIRT**	n p-value^†^	*n* = 139		*n* = 139			*p* = 1.000	*n* = 139			*p* = 1.000
Yes		4	(2.9)	4	(100.0)	0	(0.0)	4	(100.0)	0	(0.0)
No		135	(97.1)	122	(90.4)	13	(9.6)	125	(92.6)	10	(7.4)
**PEI**	n p-value^†^	*n* = 139		*n* = 139			*p* = 1.000	*n* = 139			*p* = 1.000
Yes		11	(7.9)	10	(90.9)	1	(9.1)	11	(100.0)	0	(0.0)
No		128	(92.1)	116	(90.6)	12	(9.4)	118	(92.2)	10	(7.8)
**Systemic therapy**	n p-value^†^	*n* = 139		*n* = 139			*p* = 0.239	*n* = 139			*p* = 0.070
Yes		24	(17.3)	20	(83.3)	4	(16.7)	20	(83.3)	4	(16.7)
No		115	(82.7)	106	(92.2)	7	(7.8)	109	(94.8)	6	(5.2)
**BMI**	n p-value^†^	*n* = 139		*n* = 139			*p* = 0.563	*n* = 139			*p* = 0.327
Low (≤ BMI 25.82 Kg/m2)		68	(48.9)	63	(92.6)	5	(7.4)	65	(95.6)	3	(4.4)
High (> BMI 25.82 Kg/m2)		71	(51.1)	63	(88.7)	8	(11.3)	64	(90.1)	7	(9.9)
**Size of the largest lesion**	n p-value^†^	*n* = 125		*n* = 125			*p* = 0.241	*n* = 125			*p* = 0.095
Low (≤ median size 3.2 cm)		63	(50.4)	59	(93.7)	4	(6.3)	61	(96.8)	2	(3.2)
High (> median size 3.2 cm)		62	(49.6)	54	(87.1)	8	(12.9)	55	(88.7)	7	(11.3)
**Overall Survival [Months]**	n p-value^§^	*n* = 133		*n* = 133			*p* = 0.373	*n* = 133			*p* = 0.565
Total / Events / Censored		133 / 50 / 83		121 / 45 / 76		12 / 5 / 7		124 / 46 / 78		9 / 4 / 5	
Median Survival		62.0		62.0		77.1		62.0		77.1	
95% C.I. (standard error)		49.9–74.1 (+-6.2)		50.0–74.0 (+-6.1)		/		50.0–74.0 (+-6.1)		/	
**Tumor Specific Survival [Months]**	n p-value^§^	*n* = 126		*n* = 126			0.701	*n* = 126			*p* = 0.792
Total / Events / Censored		126 / 21 / 105		114 / 19 / 95		12 / 2 / 10		117 / 20 / 97		9 / 1 / 8	
Median Survival		n.c.		n.c.		n.c.		n.c.		n.c.	
95% C.I. (standard error)		n.c.		n.c.		n.c.		n.c.		n.c.	
**Sorafenib Overall Survival [Months]**	n p-value^§^	*n* = 18		*n* = 18			*p* = 0.673	*n* = 18			*p* = 0.673
Total / Events / Censored		n.c.		14 / 7 / 7		4 / 2 / 2		14 / 7 / 7		4 / 2 / 2	
Median Survival		n.c.		48.0		19.2		48.0		19.2	
95% C.I. (standard error)		n.c.		16.9–79.1 (+-15.9)		n.c.		16.9–79.1 (+-15.9)		n.c.	
**Sorafenib Tumor Specific Survival [Months]**	n p-value^§^	*n* = 17		*n* = 17			0.701	*n* = 17			*p* = 0.930
Total / Events / Censored		n.c.		13 / 5 / 8		4 / 1 / 3		13 / 5 / 8		4 / 1 / 3	
Median Survival		n.c.		48.0		n.c.		48.0		n.c.	
95% C.I. (standard error)		n.c.		12.3–83.7 (+-18.2)		n.c.		12.3–83.7 (+-18.2)		n.c.	

† Fisher’s exact test, ‡ Kendall’s tau test, § Log-rank test. * p values having lost significance according to the Siemes (Benjamini-Hochberg) procedure for multiple testing. Abbreviations: n.c., not calculated; MASLD, metabolic dysfunction associated steatotic liver disease; BCLC, Barcelona Clinic Liver Cancer staging system; UCSF, University of California San Francisco criteria; AFP positive max. 400, AFP values below or above 400 ng/ml; TACE, transarterial chemoembolisation; SIRT, Selective Internal Radiation Therapy; PEI, percutaneous ethanol injection.

### Immunohistochemical detection of IR and IGF1R in HCC tissues

**Fig. 1:**
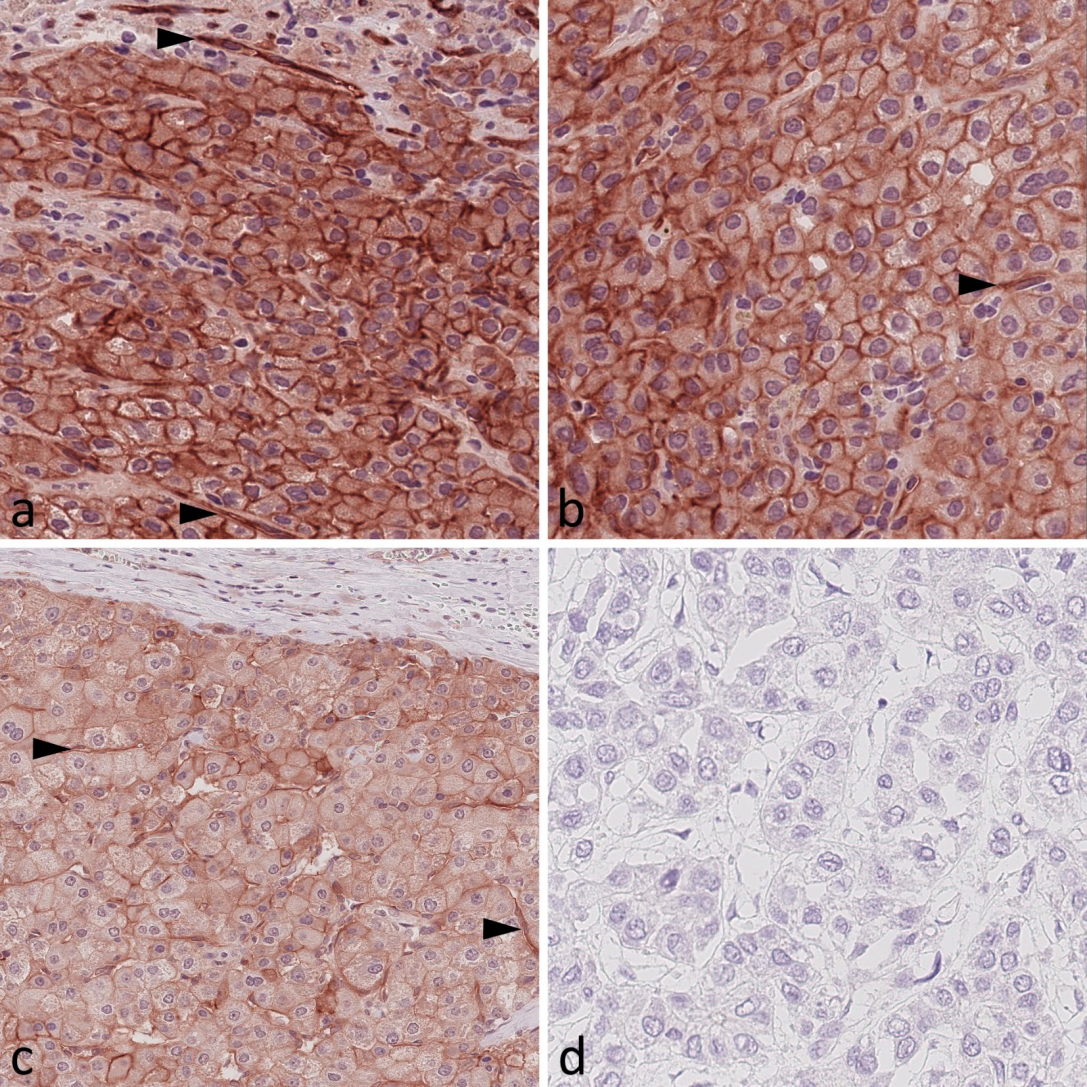
Expression of the insulin receptor in hepatocellular cancer tissue. Representative hepatocellular cancer tissue samples showing (**A**) strong membranous (mCC-IR 2+), strong cytoplasmic (cCC-IR 2+) and strong vascular (arrow heads, VIR 2+) insulin receptor expression, (**B**) weak cytoplasmic (cCC-IR 1+) and strong cytoplasmic (cCC-IR 2+), weak (mCC-IR 1+) as well as strong membranous (mCC-IR 2+) insulin receptor expression, weak vascular (arrow head, VIR 1+) insulin receptor expression, (**C**) weak (mCC-IR 1+) membranous, weak cytoplasmic (cCC-IR 1+) and weak vascular (VIR 1+, arrowheads) insulin receptor expression and (**D**) absent tumoral or vascular insulin receptor expression. Anti-insulin receptor immunostaining, hematoxylin counterstaining. Original magnification A-D: 400x.

**Fig. 2:**
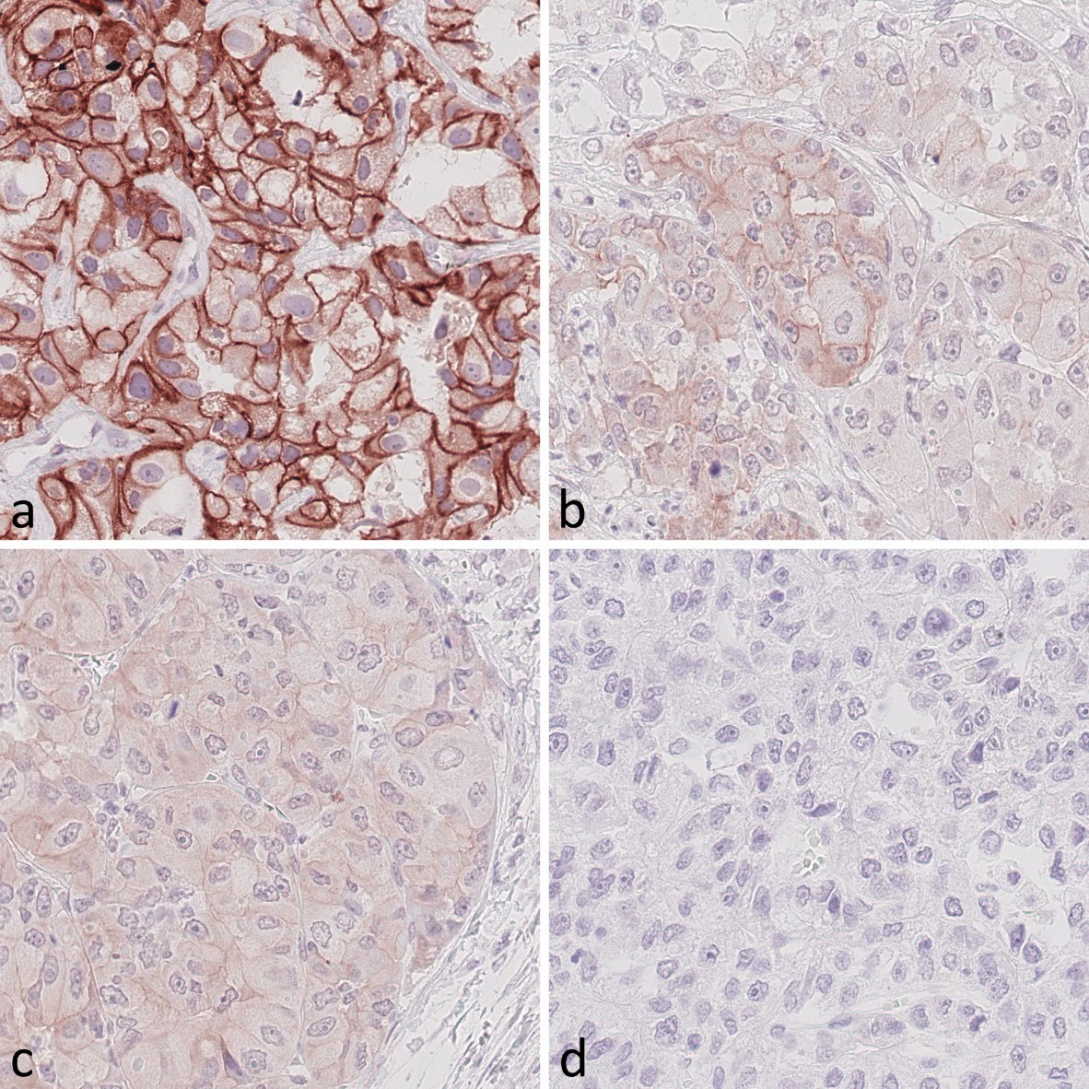
Expression of the IGF1 receptor in hepatocellular cancer tissue. Representative hepatocellular cancer tissue samples showing (**A**) strong membranous (m-IGF1R 2+) as well as predominantly weak cytoplasmic (c-IGF1R 1+) and locally strong cytoplasmic (c-IGF1R 2+) IGF1 receptor expression in tumor cells, (**B**) weak cytoplasmic (c-IGF1R 1+) and locally weak membranous (mIGF1R 1+) IGF1 receptor expression, (**C**) showing weak cytoplasmic (c-IGF1R 1+) IGF1 receptor expression and (**D**) no sign of IGF1 receptor expression (IGF1R 0). Anti-insulin growth factor 1 immunostaining, hematoxylin counterstaining. Original magnification A-D: 400x.

Whole tissue sections and biopsy specimens were used to study IR and IGF1R expression in HCC. The evaluation of IR immunostaining included the assessment of membranous (mCC-IR) and cytoplasmic (cCC-IR) expression in cancer cells, as well as vascular (VIR) IR expression in cancer vasculature. The evaluation of IGF1R immunostaining encompassed the examination of membranous (m-IGF1R) and cytoplasmic (c-IGF1R) IGF1R expression in cancer cells. Three possible staining intensities were distinguished and were observed in varying combinations and extents within one respective sample (Figs. 1 and 2). mCC-IR, cCC-IR, VIR, m-IGF1R and c-IGF1R were found to be heterogeneously expressed within the cancer tissue.

Tumor cells displayed strong cytoplasmic immunostaining (cCC-IR 2) in 35 (25.2%) and weak cytoplasmic immunostaining (cCC-IR 1+) in 118 (84.9%) cases. Tumor cells lacking any cytoplasmic IR immunostaining (cCC-IR 0) were seen in 21 (15.1%) cases. The median HistoScore (HScore) for cCC-IR was 39 (range 0-175) and the study cohort was dichotomized into cCC-IR high (HScore > 39) and cCC-IR low (HScore ≤ 39). Dichotomization discerned 70 (50.4%) cases as cCC-IR high and 69 (49.6%) as cCC-IR low.

Cancer cells were seen to have strong membranous IR immunostaining (mCC-IR 2+) in 83 (59.7%) and weak membranous IR immunostaining (mCC-IR 1+) in 116 (83.5%) samples. 22 (15.8%) cases were devoid of any membranous IR immunostaining (mCC-IR 0). The median HScore for mCC-IR was 35 (range 0–200). The study collective was dichotomized into mCC-IR high (HScore ≥ 35) and mCC-IR low (HScore < 35). 70 (50.4%) HCC samples were classified as mCC-IR high and 69 (49.6%) samples as mCC-IR low.

CD31 immunostaining confirmed the presence of cancer capillaries in all tumor sections, which was the precondition for further assessment of VIR expression.

VIR expression was confined to the tumor site and was not observed within the surrounding non-neoplastic tissue. VIR was primarily observed in capillaries and only to a lesser extent in arterioles or venules. VIR was found to exhibit strong immunostaining (VIR 2+) in 123 (88.5%) and weak immunostaining (VIR 1+) in 123 (88.5%) cases, which demonstrates that different staining intensities were regularly simultaneously present within the respective samples. 10 (7.2%) cases were devoid of any vascular IR immunostaining (VIR 0). The median HScore for VIR was 120 (range 0-200), which allowed for dichotomization into VIR high (HScore > 120) and VIR low (HScore ≤ 120). 68 (48.9%) cases were classified as VIR high and 71 (51.1%) as VIR low.

Cancer cells with strong cytoplasmic IGF1R immunostaining (c-IGF1R 2+) were observed in only 2 (1.4%) and cancer cells with a weak cytoplasmic immunostaining (c-IGF1R 1+) were seen in 13 (9.3%) cases. 126 (90.6%) samples lacked any c-IGF1R expression. The median HScore for c-IGF1R was 0 (range 0–70). The study collective was dichotomized for c-IGF1R expression into c-IGF1R low (HScore 0) and c-IGF1R high (HScore > 0). Consequently all 13 (9.4%) cases with immunohistochemical c-IGF1R expression were classified as c-IGF1R high and the remaining 126 (90.6%) cases devoid of c-IGF1R immunostaining were classified as c-IGF1R low/negative.

Tumor cells were seen to have strong membranous IGF1R immunostaining (m-IGF1R 2+) in 7 (5.0%) cases and weak membranous IGF1R immunostaining (m-IGF1R 1+) in 9 (6.5%) cases. Samples devoid of any membranous IGF1R immunostaining (m-IGF1R 0) were observed in 129 (92.8%) cases. The median HScore for m-IGF1R was 0 (range 0–76) and was utilized for dichotomization into m-IGF1R low/negative (HScore 0) and m-IGF1R high (HScore > 0). The 10 (7.2%) samples with m-IGF1R expression were thus defined as m-IGF1R high and those 129 (92.8%) samples without mIGF1R expression as m-IGF1R low/negative.

### Correlation of insulin receptor and IGF1 receptor expression in cancer cells and vessels in HCC tissues

cCC-IR high correlated significantly with mCC-IR high (*p* < 0.001) and c-IGF1R high was significantly associated with m-IGF1R high (*p* < 0.001), both of which was confirmed by multiple testing. VIR high was significantly associated with mCC-IR high (*p* = 0.028), which lost its significance upon correction for multiple testing. cCC-IR high and VIR high were not associated (*p* = 0.091). No significant correlations were found between the IR and IGF1R expression status (supplementary information, Table S1).

### Correlation of insulin receptor expression with clinicopathological patient characteristics

In order to evaluate the potential clinical role of IR expression in HCC we correlated mCC-IR, cCC-IR and VIR expression with clinicopathological patient characteristics (Table 1). mCC-IR high was significantly associated with the administration of systemic therapy in HCC (*p* = 0.012) and with less hepatic resections (*p* = 0.042). Significance was lost upon correction for multiple testing. cCC-IR high and VIR high were not associated with clinicopathological patient characteristics.

### Correlation of IGF1 receptor expression with clinicopathological patient characteristics

c-IGF1R high was associated with AFP positivity (*p* = 0.037) and the diagnosis of diabetes mellitus (*p* = 0.034). Correction for multiple testing led to the loss of statistical significance. No further significant associations between clinicopathological patient characteristics and either m-IGF1R or c-IGF1R were found (Table 2).

### Survival analysis

**Fig. 3:**
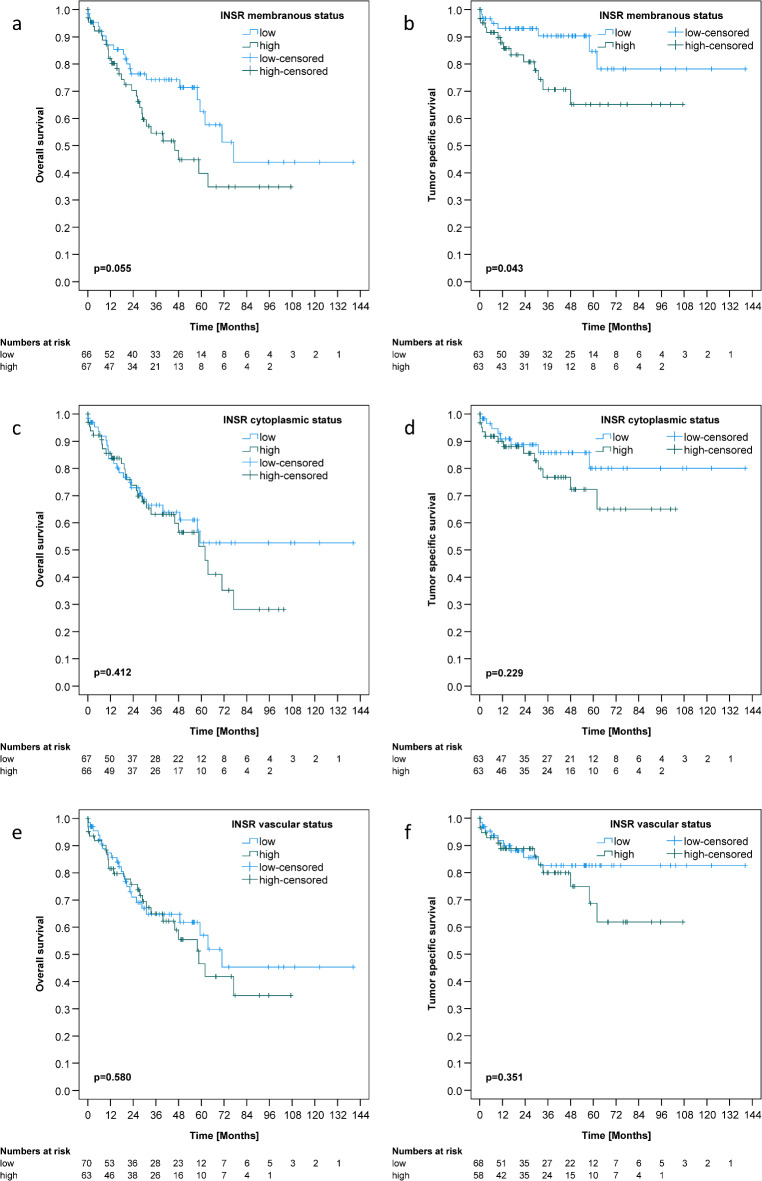
Insulin receptor expression and survival of hepatocellular cancer patients. The Kaplan-Meier curves illustrate the correlations between membranous insulin receptor expression in tumor cells (mCC-IR) and overall (**A**) (*p* = 0.055) as well as tumor specific survival (**B**) (*p* = 0.043). The Kaplan-Meier curves depict correlations between cytoplasmic insulin receptor expression in cancer cells (cCC-IR) and overall (**C**) (*p* = 0.412) as well as tumor specific survival (**D**) (*p* = 0.229). The Kaplan-Meier curves show the correlations between insulin receptor expression in tumor vasculature (VIR) and overall (**E**) (*p* = 0.580) as well as tumor specific survival (**F**) (*p* = 0.351).

The median overall survival (OS) of the HCC collective was 62.0 months. The median tumor specific survival (TSS) could not be calculated, as less than half of the patients had died a tumor-related death at the time of our statistical analysis. The mean TSS was 109.4 months. mCC-IR high was significantly associated with diminished TSS (*p* = 0.043) (Fig. 3).

**Fig. 4:**
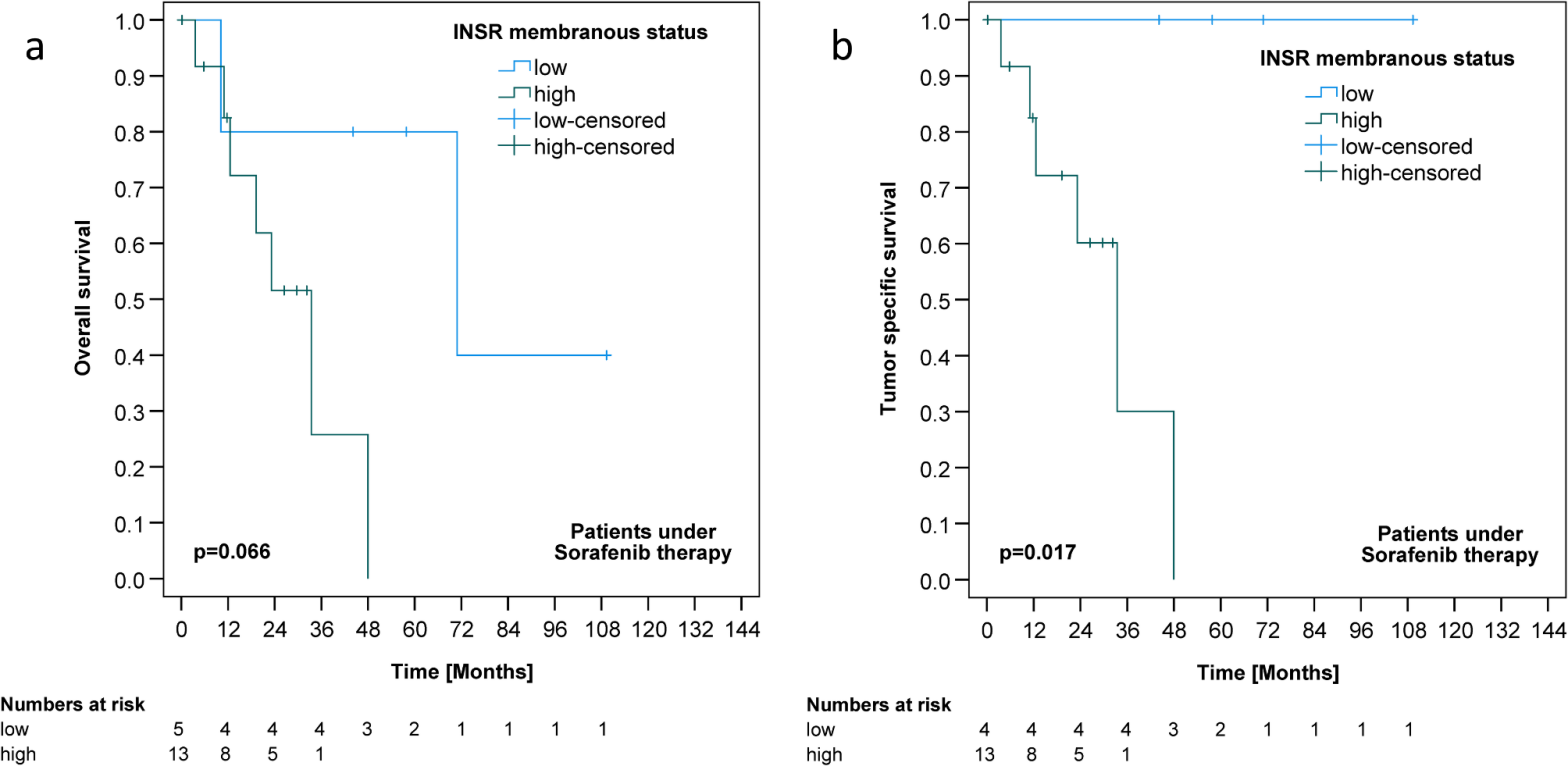
Insulin receptor expression and survival of hepatocellular cancer patients undergoing sorafenib therapy. Kaplan-Meier curves demonstrating correlations between membranous insulin receptor expression in tumor cells (mCC-IR) and overall (**A**) (*p* = 0.066) as well as tumor specific survival (**B**) (*p* = 0.017) in patients undergoing sorafenib therapy.

When focusing on the group of HCC patients undergoing sorafenib therapy, mCC-IR high was significantly associated with worse TSS (*p* = 0.017) (Table 1; Fig. 4). Significance was lost after correction for multiple testing. cCC-IR, VIR, c-IGF1R and m-IGF1R status were not associated with survival (Tables 1 and 2; Fig. 3 and S2).

On multivariate analysis, we identified the vascular invasion status (*p* = 0.001, HR = 4.91; 95%-confidence interval 1.92–12.55 months) and the resection status (*p* < 0.001, HR = 15.41; 95%-confidence interval 3.91–60.81 months) to be independent prognosticators of OS.

### Insulin and IGF1 receptor expression in hepatocellular adenomas and non-neoplastic liver specimens

10 hepatocellular adenomas (HCA) and 5 non-neoplastic non-cirrhotic healthy liver specimens were tested for IR and IGF1R expression. As the overall number of samples was low, we decided not to use the median for statistical analysis, but to rank the samples according to their HScores for further comparison (supplementary information, Table S2; Fig. S1). The mean rank of the membranous IR expression HScores of tumor-free liver tissue (range 15–82; median HScore = 57) was significantly higher than the mean rank of adenoma specimens membranous IR expression HScores (range 0–35; median HScore 5) (*p* = 0.017). Significance was lost upon correction for multiple testing. No differences between healthy liver and adenoma tissues were found with regard to cytoplasmic or vascular IR-expression, or with regard to membranous or cytoplasmic IGF1R expression.

## Discussion

The therapeutic management of HCC is in need of new prognosticators, especially in the setting of an advanced disease at diagnosis. Preclinical studies describe a role of the IR-/IGF1R-axis in HCC^9–11^, early hepatocarcinogenesis^11^ and HCC progression^12^, but real-world clinical data are needed. Therefore, we investigated in our cohort of HCC patients whether the IR or the IGF1R could be associated with clinicopathological parameters and whether their expression is prognostically significant. Our study of the IR / IGF1R expression profile in HCC provided intriguing results and revealed new aspects of HCC. The median age of our study cohort and the predominance of male patients corresponded to the demographics of other European real-world HCC study cohorts^13^.

While IR overexpression was frequently observed in our HCC collective, IGF1R expression was rarely seen in HCC specimens. Thus, IGF1R signaling does not appear to play a role in most HCCs. Although IGF1R-expression was associated with diabetes mellitus at first, the association lost its significance after correction for multiple testing. However, as the focus of the accessible patient files laid on HCC diagnosis and treatment, metabolic co-morbidities such as diabetes mellitus were only infrequently documented. It is known that IGF and insulin levels are chronically increased in patients with diabetes mellitus, which is suspected to promote carcinogenesis^14^, which has been shown for patients with cirrhosis from nonalcoholic fatty liver disease^15^. A limitation might be the fact that in our collective almost one third of all HCCs had been diagnosed in non-cirrhotic liver, which could be indicative of a diagnostic bias potentially leading to an underrepresentation of liver cirrhosis. Nevertheless, a possible association between diabetes mellitus and IGF1R expression in HCC should be further investigated in future studies.

The spatial and intracellular distribution profile of IR expression was characterized in our study. Although we did not perform a PCR-analysis of the HCC specimens to distinguish between both IR isoforms, it is known that the isoform IR-A is overexpressed in HCC^10,16^, in contrast to physiological liver tissue. We frequently observed IR overexpression in HCC cells as well as in cancer vasculature. We speculate that IR overexpression in HCC might be caused by hypoxia, which is known to promote HCC progression: Roudnicky et al. demonstrated in bladder cancer that hypoxia upregulated IR expression *in vitro.*^17^ IR upregulation was mediated by the hypoxia-inducible factor-1α (HIF-1α). In the case of HCC, increased HIF-1α expression has been described to be associated with worse survival and advanced tumor stages by several study groups^18–20^. This potential link to IR expression would fit to our observation of a diminished survival of IR overexpressing HCC patients.

We found that membranous and not cytoplasmic IR expression was associated with survival. mCC-IR overexpression was predominantly found in HCC patients who received a palliative systemic therapy and significantly less in patients who had been admissible for surgical therapy, which is suggestive for an association between mCC-IR overexpression and an advanced stage at diagnosis. As membranous IR expression has been described to be influenced by the concurrent co-expression of other transmembrane receptors^21^, future studies are necessary to expose the receptor co-expression profile of mCC-IR expressing HCC specimens. The notion of mCC-IR expression being a new prognostic biomarker in HCC biology is supported by the observation of a significantly reduced TSS. The classically calculated scores in HCC therapeutic management, such as the BCLC, UCSF or Milan scoring systems, were not indicative of the IR expression status. A limitation is of course the study’s retrospective design. mCC-IR overexpression should be further prospectively evaluated in larger patient cohorts as a prognostic biomarker for HCC survival that appears to be independent of conventional scoring systems.

When focusing on the subgroup of HCC patients undergoing sorafenib therapy, mCC-IR high was significantly associated with worse TSS, which implies that IR overexpression might be associated with mechanisms of sorafenib therapy resistance. A limitation is the limited number of patients in this subgroup, which requires future validation of our findings. We speculate that the association between mCC-IR high and the diminished survival of HCC patients undergoing sorafenib therapy might be linked by hypoxia-induced drug resistance. Hypoxia of the tumor microenvironment has been shown to lead to sorafenib resistance in HCC patients^22^. It has been described in preclinical experiments that an inhibition of hypoxia-inducible factors (HIF) can overcome sorafenib resistance^22^. We therefore hypothesize that mCC-IR high might serve as a predictive biomarker of hypoxia-induced drug resistance in HCC. As therapy resistance to lenvatinib is also known to be associated with hypoxia^23,24^, we hypothesize that mCC-IR high could potentially serve as an indicative biomarker for the treatment response of a therapy with lenvatinib as well.

In recent years, the systemic treatment of HCC has changed rapidly. In the modern treatment of advanced HCC, the combination of the checkpoint inhibitor atezolizumab and the VEGF inhibitor bevacizumab, or, alternatively, combined checkpoint inhibition with tremelimumab and durvalumab are currently the first choice for patients with an ECOG (Eastern Cooperative Oncology Group Performance) 0–1 status who are classified as Child Pugh A^25^. If there is a contraindication to the two therapy combinations described, therapy with sorafenib, lenvatinib, or durvalumab monotherapy is recommended^25^. In patients with advanced HCC, a Child-Pugh stage B and a good performance status, checkpoint inhibition with an anti-PD1/anti-PD-L1 antibody or the tyrosine kinase inhibitors sorafenib or lenvatinib may be offered as first-line therapy after individualized assessment according to the ASCO guidelines^25^. A limitation of our study is the absence of patients treated with modern immunotherapy regimens.

Tumor hypoxia has also been linked to therapeutic resistance to checkpoint inhibition^26^. Whether IR overexpression may also correlate with resistance to checkpoint inhibition remains to be validated in future studies. With this in mind, future studies will need to assess whether HCC patients (Child Pugh A / ECOG 0–1) with mIR overexpression might benefit more from a diversified therapeutic approach, such as the therapeutic combination of atezolizumab with bevacizumab, which does not rely solely on the principle of checkpoint inhibition. With respect to patients with an advanced HCC and a Child-Pugh stage B, future studies are needed to evaluate if the mIR overexpression status could serve as a predictive biomarker to opt for or against a tyrosine kinase inhibitor therapy in this subset of vulnerable patients.

Despite established immuno-oncology therapy, tyrosine kinase inhibitors still have clinical relevance in the treatment of HCC, as recently demonstrated in a real-world study^27^. Future studies of IR overexpression and treatment resistance should include additional experimental models. Experimental models of IR-overexpressing HCC should be used to assess treatment resistance to tyrosine kinase inhibitor-based therapies compared to immunotherapies and to investigate the underlying mechanisms that drive therapeutic resistance.

In conclusion, membranous IR overexpression was associated with worse survival in HCC. The subset of HCC patients with a positive membranous IR expression status did not benefit from tyrosine kinase inhibitor therapy with sorafenib. Future studies are needed to further validate this finding. If membranous IR overexpression could be used as a predictive biomarker to opt for or against a tyrosine kinase inhibitor therapy, this would be particularly useful for therapeutic decision-making in the vulnerable subgroup of patients with advanced HCC and Child-Pugh stage B. The IR expression status has the potential to refine therapeutic decision-making in HCC therapy and should therefore be further investigated.

## Materials and methods

### Study population and histology

From the archive of the Department of Pathology, University Hospital Schleswig-Holstein, Kiel, Germany, we retrieved all available specimens from HCC patients of the Liver Transplant Department and the Hepatology Department of the University Hospital Schleswig-Holstein (UKSH) Kiel, Germany. The tissue specimens originated from diagnostic biopsies, therapeutic resection specimens or liver explants between 2004 and 2018. As the extent of a sample size determines the robustness of results, we had decided not to limit the sample size, but to include all accessible HCC specimens which met the inclusion and exclusion criteria. Patients were included if an HCC had been confirmed by histology. Samples were excluded if a tumor type other than HCC was identified, or if the paraffin block did not contain sufficient remaining non-necrotic tissue.

Gross sectioning and histological examination were performed by board certified and trained surgical pathologists. Data about the date of patient death and the cause of death were provided by the Epidemiological Cancer Registry of the state of Schleswig-Holstein, Germany. All patient data were pseudonymized after study inclusion.

The study was conducted according to the guidelines of the Declaration of Helsinki, and approved by the Ethics Committee of the University Hospital Schleswig-Holstein, Kiel, Germany (D 502/18; 2018), which allowed us to use the patient material. Informed consent was obtained from all subjects involved in the study. All patients had given written informed consent for a possible future scientific use of their biological material before the respective procedures.

### Histology

Tissues were fixed in neutral buffered formalin followed by paraffin embedding. All specimens were sectioned and deparaffinized. Hematoxylin and eosin staining was performed. Histological classification was based on the World Health Organization criteria. The pTNM stage of all patients in the study was determined according to the 8th edition of the UICC guidelines^28^ in accordance with the corrected reprint of the year 2020^29^.

### Immunohistochemistry

In the past, our study group has investigated the expression of the IR and the IGF1R in a variety of gastrointestinal cancers^4–7,30^. To achieve comparability between the study results and the gastrointestinal entities studied, we were careful to perform immunochistochemistry and the evaluation of our immunostaining in the same manner^5,6,30^. The methodology is therefore necessarily overlapping:

The autostainer Bond™ Max System (Leica Microsystems GmbH, Wetzlar, Germany) was used for immunohistochemistry with a monoclonal antibody directed against CD31 (dilution 1:100; mouse monoclonal antibody; JC70; Cell Marque, California, USA) according to the manufacturer`s instructions. The ER2 buffer (EDTA-buffer Bond pH 9.0) was used for antigen retrieval. The immunoreaction was carried out with the Bond™ Polymer Refine Detection Kit (DS 9800; brown labelling; Novocastra; Leica Microsystems GmbH, Wetzlar, Germany).

IR and IGF1R immunostaining were both performed manually. For IR immunostaining a rabbit monoclonal anti-insulin receptor β-antibody (dilution 1:50; clone 4B8; Cell Signaling Technologies, Danvers, USA) was used, which detects both IR isoforms, for IGF1R immunostaining a rabbit monoclonal IGF1-receptor β antibody (dilution 1:50; clone D406W; Cell Signaling Technologies, Danvers, USA) was employed. Primary antibody incubation was carried out overnight at 4 °C. Identical immunostaining protocols were performed for both immunostaining reactions:

First all sections were deparaffinized and afterwards boiled in EDTA buffer (pH 9.0; 1 min; 125 °C), then washed with Tris-buffered saline (TBS) and then treated with hydrogen peroxide block (Thermo Fisher Scientific) for 15 min, washed with TBS and then blocked with Ultra V Block (Thermo Fisher Scientific) for 5 min. The ImmPRESS reagent peroxidase universal anti-mouse/rabbit IgG – MP-7500 and the ImmPact NovaRed peroxidase substrate SK-4805 Kit (Vector Laboratories, Burlingame, CA, USA, respectively) were used for the visualization of immunoreactions. Afterwards counterstaining with hematoxylin was carried out. The omission of the primary antibody was used for negative control. Healthy endometrium samples (proliferative phase) served as positive controls.

The DM1000 microscope (Leica Microsystems GmbH, Wetzlar, Germany) was used to image and evaluate all immunostained slides. The Leica SCN400 microscopic whole-slide scanner (Leica Biosystems, Nussloch, Germany) was employed to scan representative tissue slides at 400 times magnification, which were viewed with the software Aperio ImageScope (Leica Biosystems, Nussloch, Germany).

### Evaluation of CD31-immunostaining

The CD31-immunostaining was conducted to verify the presence of cancer vasculature, particularly capillaries, within the respective tissue specimens. The capillaries, arterioles and venules, which were surrounded by HCC cancer cells, were referred to as cancer vasculature.

### Evaluation of IR and IGF1R immunostaining

A modified HistoScore (HScore) was utilized to assess the immunostaining of the IGF1R and the IR, respectively: The first step assessed the staining intensity of the respective cells. The scoring system differentiated between 0 (no staining), 1+ (weak) and 2+ (strong immunostaining evident). Secondly, the percentage of cells exhibiting no (0), weak (1+) or strong (2+) immunostaining was estimated. The percentages for each HCC sample were totaled to reach 100%. A specimen with strong immunostaining (2+) in all cancer cells would be classified as 100% “2+”. A sample with strong immunostaining (2+) in one half and absent immunostaining (0) in the other half of the sample would be classified as 50% “2+” and 50% “0”. The HScore was calculated in accordance with the following formula: HScore = [0 x percentage of immunonegative tumor cells] + [1 x percentage of weakly stained tumor cells] + [2 x percentage of strongly stained tumor cells]. In the event that all cancer cells within a tumor sample exhibited strong immunostaining, the maximal HScore of 200 would be reached in accordance with the formula: [0 × 0%] + [1 × 0%] + [2 × 100%] = 200. In the event of weak immunostaining in 30% of all cancer cells, the resulting HScore would be [0 × 0%] + [1 × 30%] + [2 × 0%] = 30. The use of multipliers enhances the stratification of the H-scores, facilitating the distinction between samples with low and high immunostaining intensities. All HCC samples were screened and representative cases were selected to serve as reference standards for IR immunostaining intensities (IR 0, IR 1 + and IR 2+) as well as for IGF1R immunostaining intensities (IGF1R 0, IGF1R 1 + and IGF1R 2+).

The IR immunostaining was evaluated for membranous (mCC-IR) and cytoplasmic (cCC-IR) expression in cancer cells, as well as for vascular (VIR) IR expression in cancer vasculature. IGF1R immunostaining was evaluated for membranous (m-IGF1R) and cytoplasmic (c-IGF1R) expression in cancer cells. It was not evaluated in vessels, as IGF1R expression had not been identified in vasculature. Finally, a median HScore was calculated for each parameter. The median HScore was used as a cut-off to distinguish between high and low IR- or IGF1R expression, respectively.

### Statistical analyses

Our study group has examined the expression of the IR and the IGF1R in several gastrointestinal cancer entities^4–7,30^ and we were careful to perform our statistical analyses in the same manner. The methodology is therefore necessarily overlapping:

For statistical analysis, we used SPSS version 24.0 (IBM Corp., Armonk, NY, USA). We tested the correlations between non-ordinal clinicopathological patient characteristics and the CC-IR status, the VIR status, or the IGF1R status with Fisher’s exact test. The ordinal scale variables of UICC stage, T category, N category and tumor grading were tested with Kendall’s tau test. The Kaplan–Meier method was used to illustrate the median survival rate with 95% confidence intervals. To test for differences between median survivals, the log-rank test was performed. We then executed a multivariate survival analysis (Cox regression). A p value of ≤ 0.05 was deemed to be significant. All p values are shown uncorrected. To account for false discovery rates within the correlations, we used the Siemes (Benjamini-Hochberg) procedure. P values that have lost significance are indicated. A multivariate survival analysis (Cox regression) was performed.

## Electronic supplementary material

Below is the link to the electronic supplementary material.

Supplementary Material 1

## Data Availability

Data is provided within the manuscript and supplementary information file.
