# Supplementary material for: Worse survival of hepatocellular cancer patients with membranous insulin receptor overexpression
Source: Sci Rep. 2025 Jan 7;15:1209. doi: 10.1038/s41598-025-85350-2 (PMC11707270; doi:10.1038/s41598-025-85350-2)
Supplement: Supplementary file 1 — Supplementary Material 1 [file 41598_2025_85350_MOESM1_ESM.pdf]

Supplementary information

Worse survival of hepatocellular cancer patients with membranous insulin receptor overexpression

Steffen Markus Heckl[1,2,3*+] , Carolin Schneider[1+] , Lukas Kercher[1] , Hans-Michael Behrens [1], Jan-Paul Gundlach[4], Alexander Bernsmeier[4], Stephan Schmidt[4] , Sandra Krüger[1] , Felix Braun[4] , Rainer Günther[3], Thomas Becker[4], Stefan Schreiber[3], Christoph Röcken[1]

[1]Department of Pathology, Christian-Albrechts-University, University Hospital Schleswig-Holstein, Kiel, 24105, Germany
[2]Department of Internal Medicine II, Christian-Albrechts-University, University Hospital Schleswig-Holstein, Kiel, 24105, Germany
[3]Department of Internal Medicine I, Christian-Albrechts-University, University Hospital Schleswig-Holstein, Kiel, 24105, Germany
[4]Department of General, Visceral, Thoracic, Transplant and Pediatric Surgery, Christian-Albrechts-University, University Hospital Schleswig-Holstein, Kiel, 24105, Germany
*steffen.heckl@uksh.de
[+]Both authors contributed equally to this work.

Supplementary table S1. Correlation between the expression of the insulin-like growth factor receptor 1 (IGF1R) and the insulin receptor (IR) in cancer cells and vasculature.

	Cytoplasmic IGF1R expression			Membranous IGF1R expression		
	**low (HScore = 0)** n (%)	**high (HScore > 0)** n (%)	**p-value†**	**low (HScore = 0)** n (%)	**high (HScore > 0)** n (%)	**p-value†**
**Vascular IR expression**						
**low (HScore < 120)**	64 (90.1)	7 (9.9)	1.000	66 (93.0)	5 (7.0)	1.000
**high (HScore > 120)**	62 (91.2)	6 (8.8)		63 (92.6)	5 (7.4)	
**Cytoplasmic IR expression**						
**low (HScore < 39)**	64 (91.4)	6 (8.6)	0.779	66 (94.3)	4 (5.7)	0.532
**high (HScore > 39)**	62 (89.9)	7 (10.1)		63 (91.3)	6 (8.7)	
**Membranous IR expression**						
**low (HScore <35)**	63 (91.3)	6 (8.7)	1.000	64 (92.8)	5 (7.2)	1.000
**high (HScore ≥ 35)**	63 (90.0)	7 (10.0)		65 (92.9)	5 (7.1)	

† Fisher's exact

Supplementary table S2. Correlation of insulin receptor (IR) and insulin-like growth factor receptor 1 (IGF1R) expression between liver adenoma and tumor free liver samples.

	Cohort	N	Mean Rank	Sum of Rank	p-value†
**Membranous IR expression**	Adenoma	10	6.10	61.00	**0.017***
	Tumor free	5	11.80	59.00	
	Total	15			
**Cytoplasmic IR expression**	Adenoma	10	6.50	65.00	0.070
	Tumor free	5	11.00	55.00	
	Total	15			
**Vascular IR expression**	Adenoma	10	7.30	73.00	0.421
	Tumor free	5	9.40	47.00	
	Total	15			
**Membranous IGF1R expression**	Adenoma	10	8.50	85.00	0.524
	Tumor free	5	7.00	35.00	
	Total	15			
**Cytoplasmic IGF1R expression**	Adenoma	10	8.50	85.00	0.524
	Tumor free	5	7.00	35.00	
	Total	15			

† Fisher's exact

* p values having lost significance according to the Siemes (Benjamini-Hochberg) procedure for multiple testing.

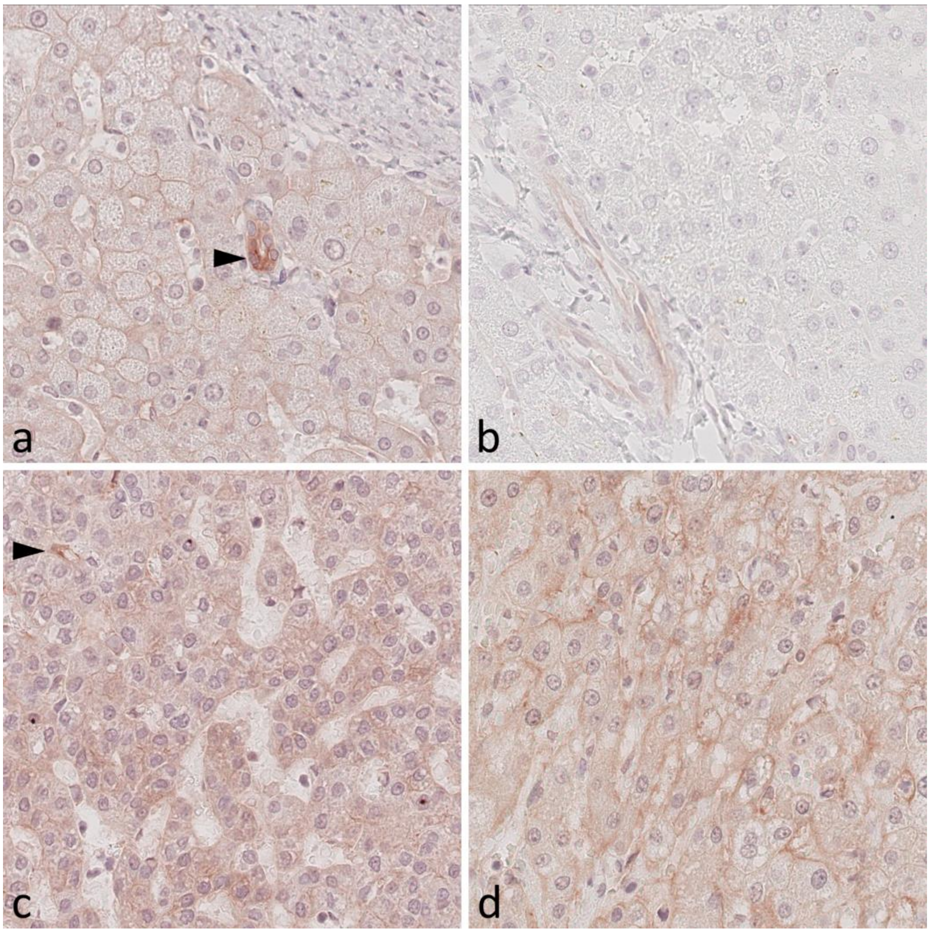

Supplementary Figure S1. Insulin receptor (IR) and insulin-like growth factor 1 receptor (IGF1R) expression in healthy liver tissue and hepatic adenomas.

Representative tissue samples showing (A) weak cytoplasmic (cCC-IR 1+) and membranous (mCC-IR 1+) IR expression in healthy liver tissue and strong cytoplasmic (cCC-IR 2+) and strong membranous (mCC-IR 2+) IR expression in a bile duct (arrow head), (B) absent IGF1 receptor expression (0) in a sample of healthy liver tissue, (C) weak cytoplasmic (cCC-IR 1+) and weak membranous (mCC-IR 1+) IR expression and strong vascular IR expression (VIR 2+) in a hepatic adenoma specimen and (D) weak cytoplasmic (c-IGF1R 1+) and weak membranous (m-IGF1R 1+) IGF1 receptor expression in a hepatic adenoma sample. Original magnification A-D: 400x.

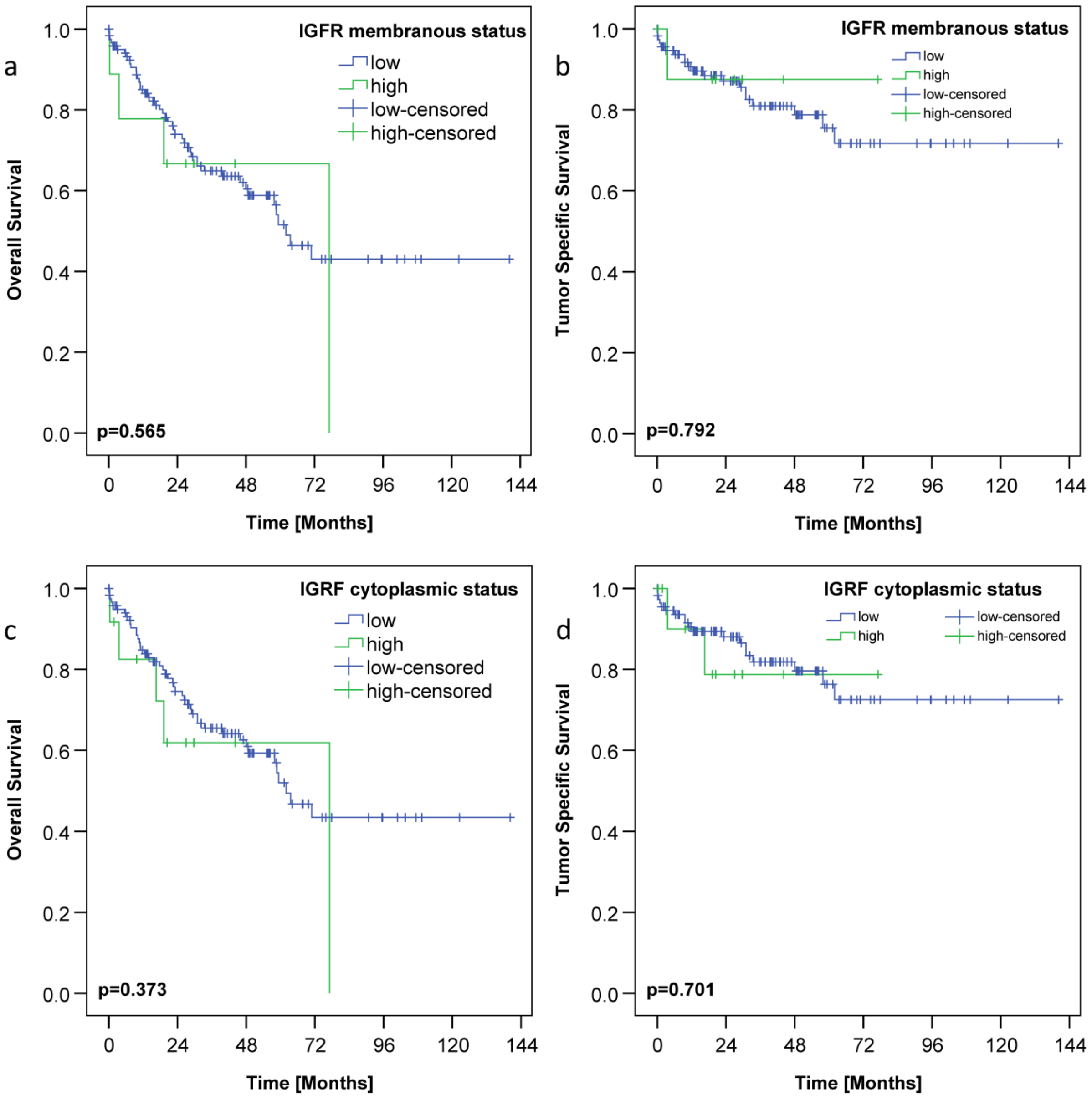

Supplementary Figure S2. IGF1 receptor expression and survival of hepatocellular cancer patients

Kaplan-Meier curves displaying correlations between membranous IGF1 receptor expression in cancer cells (m-IGF1R) and overall (A) ($p = 0.565$) and tumor specific (B) ($p = 0.792$) survival. Kaplan-Meier curves presenting correlations between cytoplasmic IGF1 receptor expression in cancer cells (c-IGF1R) and overall (C) ($p = 0.373$) and tumor specific survival (D) ($p = 0.701$).
